# Supplementary material for: Multiphysics pharmacokinetic model for targeted nanoparticles
Source: Front Med Technol. 2022 Jul 15;4:934015. doi: 10.3389/fmedt.2022.934015 (PMC9335923; doi:10.3389/fmedt.2022.934015)
Supplement: Supplementary file 1 [file Data_Sheet_1.PDF]

Supplementary Material

1 SUPPLEMENTARY TABLES AND FIGURES

1.1 Figures

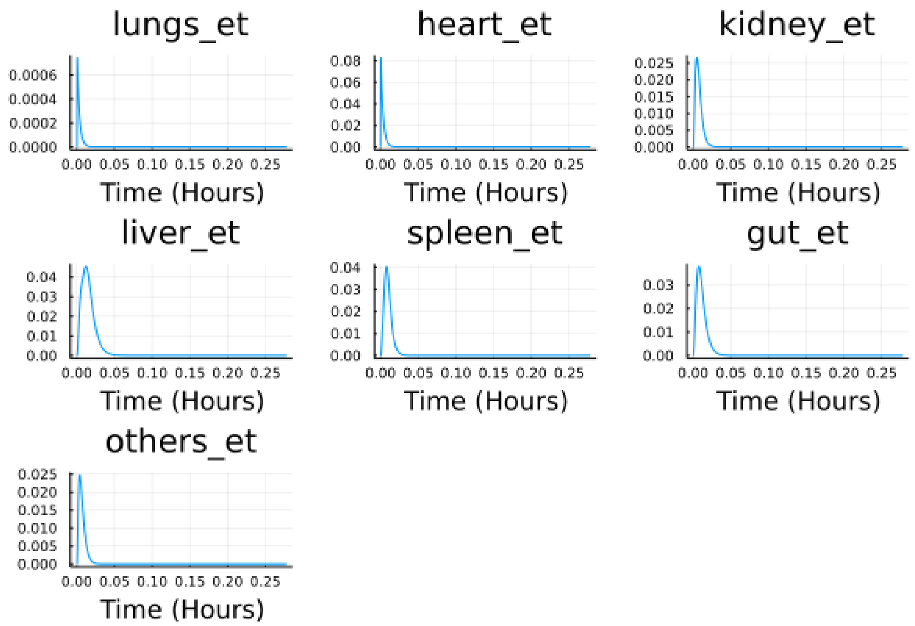

Figure S1. Molar profile of NP bound to endothelial layer for branched model. The moles of NP bound drop to zero rapidly, making it a suitable system of QSSA.

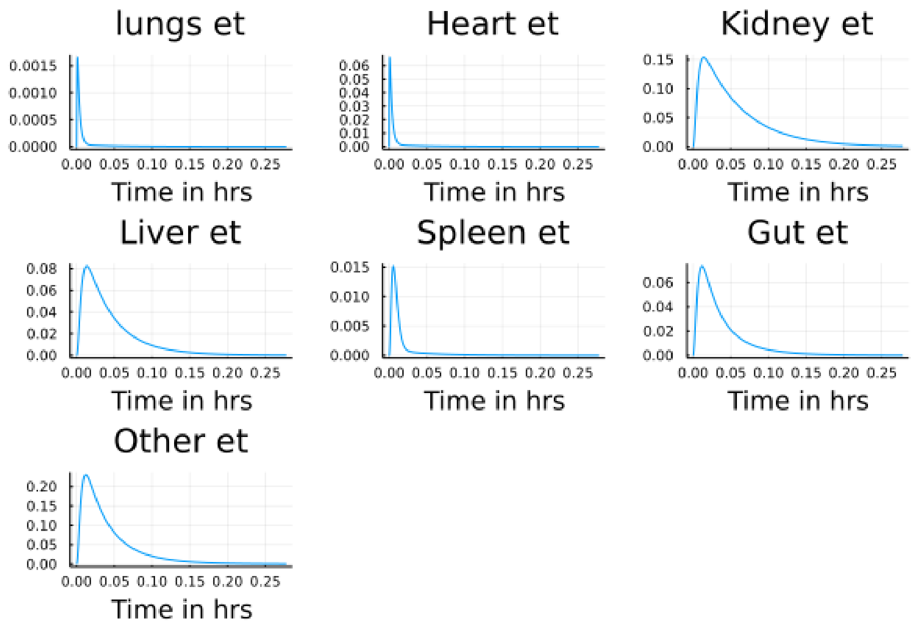

Figure S2. Molar profile of NP bound to endothelial layer for branched model.

1.2 Tables

a (nm)	Lungs	Heart	Kidney	Liver	Spleen	Gut	Others
4	3022.51	3022.51	3022.51	3022.51	3022.51	3022.51	3022.51
15	806.0	806.0	806.0	806.0	806.0	806.0	806.0
50	241.8	241.8	241.8	241.8	241.8	241.8	241.8
79	153.04	153.04	153.04	153.04	153.04	153.04	153.04
100	120.9	120.9	120.9	120.9	120.9	120.9	120.9

Table S1. K_{on} values for different organs as a function of NP diameter

a (nm)	Lungs	Heart	Kidney	Liver	Spleen	Gut	Others
4	33.8	1277.38	958.77	144.15	70.43	106.98	106.98
15	8.93	340.64	200.97	36.93	18.41	27.69	27.69
50	2.6	102.19	35.87	9.85	5.2	7.59	7.59
79	1.61	64.68	17.0	5.71	3.14	4.49	4.49
100	1.25	51.1	11.36	4.25	2.4	3.38	3.38

Table S2. K_{off} values for different organs as a function of NP diameter

a (nm)	Lungs	Heart	Kidney	Liver	Spleen	Gut	Others
4	89.43	2.37	3.15	20.97	42.91	28.25	28.25
15	90.29	2.37	4.01	21.83	43.77	29.11	29.11
50	93.02	2.37	6.74	24.56	46.5	31.84	31.84
79	95.28	2.37	9.0	26.82	48.76	34.1	34.1
100	96.92	2.37	10.64	28.46	50.4	35.74	35.74

Table S3. $log(K_{EC})$ values for different organs as a function of NP diameter

	Lung	Heart	Kidneys	Liver	Spleen	Gut	Other	Arteries	veins
K$_{deg}ModelA$	2x10^{-6}	2x10^{-6}	0.002	5x10^{-5}	5x10^{-6}	N/A	N/A	N/A	N/A
K$_{up}ModelA$	1000	0.0059	1x10^{-3}	100	0.0556	N/A	N/A	N/A	N/A
K$_{NS}ModelA$	1000	0.01	1x10^{-3}	100	0.05	N/A	N/A	N/A	N/A
K$_{deg}ModelB$	2x10^{-8}	2x10^{-6}	0.002	5x10^{-6}	1x10^{-6}	3.3x10^{-6}	3.3x10^{-4}	1.67x10^{-6}	1.67x10^{-6}
K$_{up}ModelB$	100	0.025	0.005	100	3	1x10^{-3}	1x10^{-3}	N/A	N/A
K$_{NS}ModelB$	100	0.025	0.0014	100	2.25	2x10^{-5}	2x10^{-5}	N/A	N/A

Table S4. K_{deg}, K_{up}, K_{NS}, rates in each organ compartment for Model A and Model B as determined via local sensitivity analysis.

	**Membrane Type**	**Lung R^2 Values**	**Total R^2 Values**
**Ramakrishnan (2016) Model**	Flat	0.569	0.764
	Membrane	0.876	0.777
	Membrane + Other Cells	0.876	0.687
	Membrane + Other Cells*	0.876	0.636
**Modified Model**	Flat	0.79768	0.78102
	Membrane	0.89524	0.91298
	Membrane + Other Cells	0.89524	0.91202
	Membrane + Other Cells*	0.89524	0.86731
**30 Minute simulation of unbranched model**		0.95867	0.84923

Table S5. R^2 value comparison between original Ramakrishnan 2016 model **?** and the modified model that incorporates non-specific NP uptake. R^2 values represent the correlation of model output to an experimental data set.

K_{on}	Stiffness Ratio
10^3	10^9
10^2	10^8

Table S6. Dependence of stiffness ratio on K_{on}
